# Exploring the Use and Effects of Deliberate Self-Harm Websites: An Internet-Based Study

**DOI:** 10.2196/jmir.2802

**Published:** 2013-12-20

**Authors:** Isobel Marion Harris, Lesley Martine Roberts

**Affiliations:** ^1^Primary Care Clinical SciencesSchool of Health and Population SciencesCollege of Medical and Dental Sciences, University of BirminghamBirminghamUnited Kingdom

**Keywords:** Internet, websites, deliberate self-harm, self-injury

## Abstract

**Background:**

In the United Kingdom, rates of deliberate self-harm (DSH) are rising. Alongside this, there has been an increase in the number of websites available with DSH content, and the Internet is known as a valuable resource for those who feel isolated by their condition(s). However, there is little and contradictory evidence available on the effects of using such websites. Further research is therefore required to examine the use and effects of DSH websites.

**Objective:**

Our objectives were to explore (1) the reasons people engage in the use of self-harm forums/websites, (2) the beliefs of users of self-harm forums regarding the role of such websites, (3) how the use of self-harm forums/websites modulates self-harm behaviors, and (4) other ways that self-harm forums affect the lives of individuals who use them.

**Methods:**

Data were collected by a questionnaire hosted on 20 websites with self-harm content. Participants were self-selected from users of these sites. Results were analyzed using descriptive statistics and simple thematic analysis.

**Results:**

In total, 329 responses were received with 91.8% (302/329) from female site users. The majority of participants (65.6%, 187/285) visited these sites at least twice per week, and most participants used the sites to find information (78.2%, 223/285) or participate in the forums (68.4%, 195/285). Positive effects of website use such as gaining help and support, isolation reduction, and a reduction in self-harm behaviors were reported by a large number of participants. However, smaller but important numbers reported negative effects including worsened self-harm, being triggered to self-harm, and additional negative physical and psychological effects.

**Conclusions:**

This is the first multisite study to explore DSH website use in depth. There are clear and important benefits to engaging in website use for many individuals; however, these are not experienced by all website users. Negative effects were experienced by moderate numbers following website use, and clinicians should consider the impact of a patient’s website use when consulting.

## Introduction

The term deliberate self-harm (DSH) can be defined as “the intentional destruction of body tissue without suicidal intent” [1]. DSH encompasses a range of behaviors including cutting, burning, scalding, hair pulling, banging body parts, breaking bones, needle-sticking, scratching, preventing wound healing, and self-poisoning [1-4], although it has been suggested that cutting is the most common behavior [5].

Prevalence of DSH in the United Kingdom has been estimated to be between 4.6% and 6.6% [6,7]; however, it is thought actual rates may be higher due to suspected levels of unreported behavior. The Adult Psychiatric Morbidity in England 2007 [7] report indicated an increase in the proportion of people reporting DSH between 2000 and 2007, particularly in women. Gender disparity is most evident in data from 16-24 year olds with 17.0% of young women self-harming compared to 7.9% of men in the same age group [7].

The Internet has been shown to be a valuable resource for individuals who feel isolated by their condition(s), such as those who self-harm, by providing anonymous support and information [8]. Internet access has increased greatly over the last decade with an estimated 80% of British households having Internet access [9]. The diversity of the Internet means it has innumerable uses, and health information has been shown to be more frequently accessed on websites than in the past [10].

Prasad et al researched the type of information and help available on the Internet for those who self-harm and concluded that the majority of websites offered information on self-harming, suicide, and related psychological issues, but that little support and discussion opportunities were available [11]. More recently, the number of Internet-based discussion pages available for self-harm has increased [12] and this, along with the increase in Internet accessibility and developments in online communication, means that opportunities for Internet-based self-harm discussion are more widely available than ever before.

Online discussion forums and health websites have generated concerns, and studies of similar websites, such as those for sufferers of eating disorders, have suggested behaviors encouraged by the use of such sites may be of concern. However, such sites are also seen as a source of support for users [13]. Previous research looking at self-harm discussion sites has concluded that there are positive aspects associated with use of these websites. For instance, evidence suggests such websites provide valuable emotional support for individuals who self-harm [8,14] and that these websites can be used as a coping mechanism for self-harm behaviors [15].

However, concerns have been raised over perceived negative effects of such websites, such as encouragement or triggering of DSH behaviors [8,14], normalization of DSH behaviors that may prevent individuals from seeking professional help [14,15], and the fact that support offered on such websites follows a very different model and style from support offered by health care professionals [14]. It has been suggested such websites could facilitate the learning of new self-harm behaviors [8].

Three main reasons for why such websites are used have been identified: users viewed them as places of support and understanding, as a community to which they could belong and as offering coping mechanisms for the social and psychological distress associated with self-harm [15]. Additionally, users of self-harm websites have reported they believe use of these sites had a positive effect on their self-harm behavior, particularly in terms of reducing frequency and severity [12].

Current evidence on this topic, however, is significantly limited by users being drawn from only one website and small sample sizes, meaning results cannot be generalized to the wider self-harm website-using population and highlighting the need for further research. Websites vary greatly in terms of intent and content, and therefore wider reaching research is required including a broader selection of the websites available.

In order to explore the use of and effects of using these websites, this study had four aims: (1) to explore the reasons people engage in the use of self-harm websites/forums, (2) to explore the beliefs of users of self-harm forums regarding the role of such websites, (3) to explore how the use of self-harm websites/forums modulates self-harm behaviors, and (4) to identify other ways that self-harm websites/forums affect the lives of individuals who use them.

## Methods

### Data Collection

This study received ethical approval from the University of Birmingham BMedSc Internal Ethics Committee prior to commencement.

All data were collected via an anonymous questionnaire developed after a review of the available literature. The questionnaire gathered data on DSH behaviors, DSH website/forum use, and reasons for use of such websites/forums, using both closed and open questions. Closed questions, where participants selected the most appropriate answer(s) from a list of options, were used where there was robust prior evidence about the subject. Open questions with free-text response were used where little was known on the subject. The format of the questionnaire was standard for all participants, and participants were able to review or change their answers once a question had been completed. Responses to the questionnaire were reviewed on the first day of posting to ensure comprehension by participants and that no changes were deemed necessary.

The questionnaire, hosted by SurveyMonkey, was accessed by participants via a link posted on websites with self-harm content (see Multimedia Appendix 1 for the questionnaire). A Google search using terms such as “self-harm forums” and “self-harm websites” identified 22 websites, of which 10 initially granted permission to post the questionnaire. In each case, permission was sought from the administrator/moderator of the sites before attempting to post the link. Where possible, the link was posted as a “sticky”, or permanent, post. If this was not possible, websites were regularly checked to ensure the link had not gotten lost in the forum and if necessary was reposted.

The questionnaire was available to access for 6 weeks between January and March 2013. Participants were invited to access the questionnaire by clicking a link and reading the participant information sheet before clicking on a second link to confirm willingness to proceed to the questionnaire and participate in the study. On completion of the questionnaire, responses were automatically sent to the SurveyMonkey account. Duplicate completions of the questionnaire were prevented by SurveyMonkey to only allow one response per IP address.

One question asked participants to list the websites they currently use. When new websites were provided as answers, these websites were investigated as possible hosts for the questionnaire as described above, to ensure that the widest selection of websites possible was included in the study. In total, the questionnaire was hosted on 20 separate websites (Multimedia Appendix 2).

### Analysis

The data collected comprised both quantitative and qualitative data. Quantitative data were used to describe the sample in terms of age, ethnicity, gender, DSH behavior, and DSH website use. Simple descriptive statistics were used to describe the frequency of DSH and types of behavior (see analysis maps in Multimedia Appendix 3).

Initial analysis of qualitative data was carried out during the data collection phase; responses were analyzed by a simple thematic coding and counting method. Coding categories were developed by a single researcher, but a subsample (n=50) was reviewed by a second researcher (LR) to confirm agreement with the coding framework and individual codes. After the data collection phase, coding categories were reviewed and collapsed to give the best thematic representation of categories to address the research questions. Given the large dataset, some indication of strength of expression of themes was possible. Researchers have chosen not to report percentages in the results section as coding infers a degree of subjectivity, and therefore quantifying of data in this way would be inappropriate. However, for some key themes an approximation of the weight of reporting participants is given.

## Results

### Description of Sample

Overall, 329 participants consented to complete the questionnaire. Due to the nature of the Internet hosting of the questionnaire, participants were free to exit the questionnaire at any point resulting in some uncompleted submissions; however, these submissions were still included in the analysis. Additionally, due to the filtering of the questionnaire, participants did not necessarily answer every question. Therefore, for questions described below, the number of participants who answered each question has been detailed (n).

### Demographics

329 participants answered the demographic questions. Of these, 91.8% (302/329) were female and the average age of the sample was 23.06 years (SD 8.62). The majority of participants reported their ethnicity as white (90.3%, 297/329). Of the remaining participants, 3.3% (11/329) reported mixed ethnicity, 1.5% (5/329) Asian, 1.5% (5/329) Chinese, 0.6% (2/329) black, and 2.7% (9/329) reported “other”. Most participants selected United Kingdom as their country of residence (69.9%, 230/329). A further 7.6% (25/329) selected Europe and 22.5% (74/329) selected “other”. Participants were invited to specify if they selected “other”, and the most commonly reported countries were Canada, Australia, and New Zealand.

### Self-Harm History

Of the 329 participants who answered the question about prior self-harm, 98.5% (324/329) reported that they had previously self-harmed. No definition of DSH was provided for this question. Instead, participants were allowed to apply their own personal definition of DSH when determining whether they had previously carried out DSH or not. Further clarification of this personal definition was sought in later questions.

Of the 5 participants with no self-harm history, 3 provided reasons for their website use. One reported use for work purposes, while 2 reported use to prevent starting self-harm behavior. Table 1 reports the methods of self-harm employed as reported by 323 participants. The most common method of self-harming was cutting with 94.7% (306/323) of participants reporting this. Of the 14.6% (47/329) of participants who selected “other” for this question and were invited to specify the methods they used, biting and overdosing were most commonly reported.

There were 321 participants who indicated the age at which they started to self-harm. The average age of starting self-harm was 14.07 years (SD 5.05); 67.9% (220/324) indicated that they were currently self-harming.

**Table 1:** Methods of self-harm used by participants^a^ (N=329).

Method	n	%
Cut self	306	94.7
Burn self	100	31.0
Scald self	21	6.5
Bang body parts	135	41.8
Pull hair	69	21.4
Scratch self	166	51.4
Prevent wound healing	133	41.2
Ingest toxic substances	42	13.0
Break bones	6	1.9
Other (please specify)	47	14.6

^a^Participants could select multiple methods to fully reflect personal self- harm history.

### Website/Forum Use

There were 295 participants who indicated when they had first looked at deliberate self-harm websites. The majority of participants reported initially looking at self-harm websites/forums *after* starting self-harming (90.5%, 267/295). Of these, 15.0% (40/267) reported looking at the websites within 1 month of starting self-harming, 10.5% (28/267) within 2 months of self-harming, 17.2% (46/267) between 2 months and 1 year of starting self-harming, and 57.3% (153/267) after self-harming for longer than 1 year.

Table 2 shows website use by participants in terms of time spent using them in an average week. Information was provided by 285 participants for how long they spent looking at/using websites in an average week. The largest group of participants reported using the websites “daily but for less than 4 hours per day” (31.6%, 90/285).

Table 3 shows the activities carried out when using the websites. 283 participants provided information about what they did when using the websites. “Viewing threads in the forum” was the most commonly reported activity (78.8%, 223/283). Of the 15.5% (44/283) of participants who selected “other” as their answer and were invited to provide more detail, the most commonly reported activities were “staffing/moderating” and “looking for pictures/images”. Generally, most participants used the websites to either find information or participate in forums.

**Table 2:** Time spent looking at or using DSH websites/forums in an average week (N=329).

Time	n	%
Daily (for longer than 4 hours per day)	20	7.0
Daily (but less than 4 hours per day)	90	31.6
2-6 times per week	77	27.0
Once per week	17	6.0
Less than once per week but more than once per month	32	11.2
Once per month	11	3.9
Less than once per month	38	13.3

**Table 3:** Activities carried out when using DSH websites/forums (N=329).

Activity	n	%
Search for information	195	68.9
View threads in the forum	223	78.8
Contribute to threads in the forum	158	55.8
Start threads in the forum	117	41.3
Chat	103	36.4
Send private messages	110	38.9
Play games	22	7.8
Other	44	15.5

### Reasons for DSH Website Use and Initial Motivators for Engagement With Sites

To explore these issues, four free-text questions were asked. Data were coded, categorized, and then conceptualized into themes based on the relationship of categories to each other. From this analysis, two main predominating themes were identified.

The theme with the highest strength of reporting was that of “help and support”. Participants spoke of the value they placed on the levels of support derived from use of the websites and how they felt this had contributed to their recovery:

It keeps me focused on recovery, placing importance on good mental health strategies and expressing my feelings. It helps me cope with self-harm thoughts or behavior. It’s a place helping me deal with day-to-day issues.

Participant 59—female, 29

“Isolation reduction and community engagement” was the theme with the second highest strength of reporting. Around half the participants described their experiences of self-harm as being highly isolating and lonely. They therefore used the websites to connect with other people who shared experiences/feelings and to reduce the sense of isolation they felt resulting from their self-harm:

They make me feel less alone and isolated, and make me realize there are other people who have similar feelings to me.

Participant 85—female, 18

Outside of these two predominating themes, the theme of “distraction and expression” was also apparent in large numbers of participants’ answers. Participants described the websites as places enabling expression of their feelings or providing distraction from carrying out self-harm behaviors:

They allow me to express myself and to gain some peer support. They’re also good distractions from self-harm thoughts, and games especially are helpful with that.

Participant 185—female, 30

Some participants also wrote about their desire to “help others”, a desire often driven by wanting to offer others the same support they had derived from website use:

I used it to ask for help and advice because I didn’t feel I could ask in my own life. I continue to use the site because I like to give back and give advice to those who post threads and are struggling just as I used to.

Participant 291—female, 21

After these themes, the theme of “triggering material/tips” was the next most commonly reported. While significantly fewer participants mentioned this theme, compared to other reasons for use/engagement, it was still relatively common with around 10% of participants reporting this. This theme included answers detailing looking for written material as well as images and artwork:

I wanted triggering. That sounds weird but I felt the harm I was doing was not bad enough and I needed to make it worse.

Participant 176—female, 19

### Do Self-Harm Websites/Forums Modulate Self-Harm Behaviors?

Some participants reported an increase in self-harm, some participants reported their self-harm decreased, and other participants reported no change in self-harm behaviors. The largest of these groups, approximately 40%, felt they now self-harmed less as a result of using the websites. The theme of “self-harm reduction” included the categories of reduced severity, reduced frequency, and website use to prevent self-harm. Participants described websites as having played a critical role in their recovery and helping them to find other, less destructive ways of managing their feelings:

It’s reduced my self-harm—I’ve learned other ways to cope, and these websites have taught me to think rationally about what I’m doing to myself. They’ve given me a new perspective on my self-injury, to view it as a coping mechanism and something I need help for.

Participant 22—female, 18

Although many reported reduced self-harm resulting from website use, just under a third of participants indicated that using the websites/forums had not affected their behaviors and that no connection existed between their Internet use and self-harm patterns:

I don’t feel using forums has innately changed my self-harm behavior. I feel the two are pretty independent.

Participant 154—female, 18

A similar number of participants indicated increased self-harm resulting from website use. The “increased self-harm” theme included the categories of increased severity, increased frequency, new methods learned, and competition with other users. Some participants reported the unhealthy nature of the websites and highlighted the comparison and competition they led to:

Using the forums leads to competitiveness. I see other’s scars, look at my own and think ‘I’m not like them, I’m not ill’ and it makes the whole situation worse.

Participant 50—female, 19

Outside of these three key areas, small numbers of participants reported, as a result of website/forum use, they either had sought external help or now took better care of their wounds.

### Other Impacts of Self-Harm Websites/Forums Use

Participants were asked whether DSH website/forum use had any other impacts for them. The majority indicated that the websites/forums had no additional effects apart from those upon self-harm behaviors. However, an important number did indicate “positive psychological effects”. This theme included categories such as improved self-esteem, reduced isolation, and feeling better able to cope. Participants spoke positively of the impact of the websites upon their lives and the key role they felt they had played in their recovery:

[The websites/forums] made me realize how much others do care, that I’m not alone, and recovery is possible.

Participant 282—female, 17

Participants also reported social effects and particularly described how the websites had facilitated the formation of new friendships and kindled positive involvement in the website/forum community:

When I was very isolated, participating in forums allowed me to feel I belonged somewhere, and have the social interaction that I was desperate for.

Participant 36—female, 23

Smaller numbers of participants detailed the perceived “negative effects” resulting from website/forum use, in terms of both physical and psychological effects. These included categories such as increased isolation, being hurt emotionally, and feeling triggered. Some participants wrote about how they felt the sites constructed unhealthy environments where the only topic of conversation was self-harm and how this could lead to feeling triggered to self-harm:

I used [the websites] as a teenager and in retrospect I can see that it made my self-harming worse because the only thing the users had in common was self-harm. It’s good to talk about it, but it was all that we talked about. I do not believe that surrounding myself with other self-harmers was good for me.

Participant 323—female, 23

### Beliefs of Users of Self-Harm Forums Regarding the Role of Such Websites

To further validate responses, participants were asked to describe in their own words the purpose of the website(s) they use to someone who does not self-harm. In line with reasons given for website use, the predominating theme in participants’ answers was “help and support”. Participants described websites as sources of valued support and information for those who personally self-harmed as well as those looking for information for friends/family members. Participants wrote passionately about support derived from such websites and how beneficial they found them to be in aiding recovery:

It’s a site aimed at supporting people who self-harm, aiming to help find ‘healthier’ ways of coping. Self-harm isn’t ‘forbidden’, and there isn’t a timescale in which you ‘have’ to stop self-harming, but the ultimate aim is that help and support helps people to stop. Moderators are watchful to make sure that people aren’t ‘comparing’ self-harm stories, and photos/images of self-harm aren’t allowed too.

Participant 58 – female, 53

Supporting wider impacts of sites, participants spoke of the “non–self-harm content” present on websites/forums used. Participants commonly reported that the websites used did not just contain information related to self-harm. They described advice relating to other mental health issues as well as general life advice found on these websites:

The website I most commonly use is a large support forum where people can get advice or support for a variety of different issues on many topics (not just self-harm). The site also has a chat room, articles and videos.

Participant 147—male, 16

Similar numbers reported the theme of “community” in their answers further evidencing the value of this to users. This included categories such as friendships, social networking, and isolation reduction. Participants described the websites as places they could turn to in order to connect with similar others who would understand their own feelings and places where they could always find someone to talk to. Participants wrote about website use as a method of reducing the isolating and lonely experience of self-harming:

[The website used] is a community of people who vary in age, profession and lifestyle related by the fact that they use self-harm as a coping mechanism, it’s NOT a teenage community where we egg each other on and share tips and photos. It’s a supportive community where we listen to each other and try to support people’s choices, some don’t want to stop self-harming, some do, and we listen and help where we can whatever the choice. It’s a community where we play games, have fun and make lifelong friends, not every conversation is dominated by self-harm, or talk of it. We are a microcosm of society, but we share a common pain, and support each other as much as we can.

Participant 118—female, 24

## Discussion

### Principal Findings

This is the first multisite study to examine the use and effects of using DSH websites. It recruited a large sample size from a broad range of websites and has produced several findings for discussion.

From the demographic questions it is evident the sample is typical compared to the self-harming population as previously described in the literature, seen in terms of gender [7] and DSH behaviors [1-5], highlighting the validity of this study. Results have been grouped into two key areas for discussion, which naturally address the two sides of the debate about DSH website impact: benefits and worsening self-harm.

### Benefits

This study’s results suggest there are several benefits of engaging in website/forum use in terms of recovery or reduction of self-harm behaviors. Analysis of responses shows that the majority of participants are using self-harm websites/forums for recovery-based reasons, either to seek help or to reduce self-harm.

The theme with the highest strength of reporting was “help and support” for both why individuals engage in website use and their beliefs regarding the role of such websites. Participants detailed how important they felt support gained from the websites is and wrote about the significant role of websites in facilitating their recovery, clearly highlighting the positive impacts of website use. This finding is in keeping with the findings of previous studies by Whitlock et al [8] and Messina et al [14] who identified such websites as providing valuable support for those who self-harm. This predominating positive theme can help to explain why, when looking at the effects of using self-harm websites, most felt their self-harm behavior(s) had improved since using the websites, in terms of both frequency and severity.

Another commonly reported theme for website use was “distraction and expression”. Participants detailed feelings of frustration and an inability to express feelings to people outside of the virtual community. The websites were described as places where participants felt they could “be themselves” and “vent” without feeling judged or stigmatized. Participants felt this enabled exploration of reasons behind their self-harm and provided alternative methods of dealing with their feelings, demonstrating the benefit of such sites in terms of reducing self-harm behaviors. Similarly, individuals described how they would use the websites instead of self-harming when they felt the urge to do so, showing how website use can be a useful distraction strategy from the desire to self-harm and for reducing self-harm.

Increased awareness and understanding of other mental health issues, such as depression and eating disorders, was commonly reported as a result of engaging in website use. This is significant as other mental health issues are highly likely to be comorbid with self-harm. The deepened understanding and awareness gained could help explain why the websites were felt to be places without judgment and stigmatization as participants had personal or first-hand experience of such issues themselves.

Additionally, there appear to be social benefits from engaging in website use. Many individuals wrote about website use as a way of forming friendships and how this reduced the isolation and loneliness they felt either as a result of or concurrent to their self-harm. This in turn led to improved self-esteem and confidence, demonstrating the positive psychological changes that can be associated with website use.

The idea of social benefits is supported by the theme of “isolation reduction and community engagement”, having the second highest strength of reporting. Many wrote of the isolating experience of self-harm and how website use was the only way of being able to communicate with others offering understanding. Similarly, many individuals reported they initially engaged in DSH website use to know they “weren’t alone” and seek other people feeling/behaving in the same way, clearly demonstrating the role of these websites in providing a community. Such findings, highlighting the importance of the virtual community among people who self-harm, are in line with previous research identifying sense of community as a key reason as to why people engage in such website use [8,15].

Some individuals reported that as a result of feeling less isolated and reassured that they weren’t the only person who self-harmed, they were encouraged to tell people outside of the virtual community and seek professional help. This shows the benefits of the online community in promoting recovery.

### Worsening Self-Harm

Despite the predominantly positive findings, some wrote that they used the websites to look for triggering material or tips. Additionally, answers revealed the competitive nature of self-harm that can be fueled through engaging in website/forum use, highlighting the dangers of website use for this vulnerable population. These findings are in line with previous research that has identified that use of such websites can result in encouragement or triggering of self-harm behaviors [8,14]. Participants wrote about how some websites did not moderate material posted, and while many indicated avoidance of these types of sites, others wrote that this was a reason for engaging in use. This highlights the need for research in this area to be inclusive in terms of websites used.

Although the sites are clearly used by some to trigger self-harm behaviors, it is interesting to note that when participants were asked when they had first looked at DSH websites, the majority reported they had viewed the websites only *after* starting self-harming. This shows that for most participants, it is not the websites themselves providing the trigger for the initiation of self-harm behaviors, although they can contribute to the maintenance or worsening of behaviors for some.

As mentioned, the majority felt that website use had either improved or not changed their self-harm behaviors; however, a significant number felt that website use had worsened their self-harm behavior. This group cannot be ignored, and answers suggest that competition between users and triggering posts by others detailing methods and extent of injuries are key factors contributing to worsened self-harm behavior.

Some individuals felt that using the websites had resulted in negative psychological effects such as increased isolation and worsened mood. These participants wrote of being shunned by other website users and being made to feel inadequate, highlighting that acceptance into virtual communities carries some of the same risks and etiquette as real communities and is a challenge for some individuals.

### Limitations

This questionnaire was solely available in English, meaning results may not be generalizable outside of the English-speaking community. Furthermore, research insurance restrictions meant this study did not include citizens from the United States. If a US citizen tried to complete the questionnaire, SurveyMonkey directed them to the end of the questionnaire after completing the demographic questions.

Participants for the study were recruited via DSH websites and forums. This may mean that the study underestimates the harmful effects of such sites as those who felt significant negative effects resulting from use may no longer do so, and they therefore would not have been able to access the questionnaire. Additionally, the results of this study suggest that the sample was highly active in terms of their use of DSH websites. We acknowledge that this may not be representative of all users of DSH websites. We also recognize that all data collected were retrospective and self-reported.

DSH websites/forums are diverse in content and ethos, and there were several sites that the questionnaire was not posted on due to restricted access or not being able to gain permission to post. Therefore, the results do not apply to all websites in existence, and other harms/benefits may exist relevant to specific sites. It should be remembered that the virtual world is constantly changing and websites are regularly created or deleted, which highlights this as an evolving area for future research. This is particularly relevant in terms of new types of sites and social media, such as Tumblr and Twitter, which many participants reported using more frequently now than the forums they previously used.

Despite these, this study gained a large and valid sample and has generated many important results and key points for discussion, demonstrating its worth. The results have implications for clinicians and from a public health perspective.

### Conclusions

Overall, the results of this study show that there are benefits to engaging in use of DSH websites/forums in terms of gaining help and support, reducing self-harm behaviors, reducing isolation, and other positive psychological changes such as improved self-esteem. However, negative aspects of website use have also been reported in terms of worsening self-harm behaviors, triggering material, and negative psychological effects such as increased isolation, highlighting the dangers of engaging in website/forum use. Clinicians should be mindful of the existence of these websites/forums when engaging with patients who self-harm, and discussion of an individual’s reasons for using such sites and the benefits/harms derived would be appropriate. If necessary, treatment plans should include strategies to maximize benefits and minimize harm derived from these sites.

Given the fluid and rapidly changing nature of the virtual world, further research into sites and the new platforms available facilitating self-harm material is recommended.

## Multimedia Appendix 1

The questionnaire.

## Multimedia Appendix 2

Websites that hosted a link to the questionnaire.

## Multimedia Appendix 3

Analysis maps.
