# Supplementary material for: Exploring the Use and Effects of Deliberate Self-Harm Websites: An Internet-Based Study
Source: J Med Internet Res. 2013 Dec 20;15(12):e285. doi: 10.2196/jmir.2802 (PMC3875893; doi:10.2196/jmir.2802)
Supplement: Supplementary file 1 [file jmir_v15i12e285_app1.pdf]

Appendix 2 – Questionnaire

Section A – About you

The first section of this questionnaire is designed to find out a bit about you which will help us to analyse the results we get. This questionnaire is anonymous so nothing you put will allow us to identify you.

1. How old are you in years?

   ____

2. What is your sex? ☐ Male ☐ Female

3.	What is your ethnicity?	
	☐ White	
	☐ Mixed	
	☐ Black	
	☐ Asian	
	☐ Chinese	
	☐ Other	
	If other please specify ____________________________________	

4.	What is your country of residence?	
	☐ U.K.	
	☐ Europe (not including U.K.)	

	☐ USA [Participants will be directed to end of survey]
	☐ Other
	If other please specify ____________________________________

Section B - History of deliberate self-harm

The second part of this questionnaire will help us to understand a bit more about your own experiences of self-harm.

5. Have you ever self-harmed?

- ☐ Yes
- ☐ No [Participants will be directed to Section C]

6. Do you currently self-harm?

- ☐ Yes
- ☐ No [Participants will be directed to question 8]

7. How often do you self-harm?

- ☐ More than once daily
- ☐ Once daily
- ☐ 4-6 times weekly
- ☐ 2-3 times weekly
- ☐ Every couple of weeks
- ☐ Approximately monthly
- ☐ Few times per year
- ☐ Less often than once per year

8. When you self-harm/self-harmed, what do/did you usually do?

- ☐ Cut yourself
- ☐ Burn yourself
- ☐ Scald yourself
- ☐ Bang body parts
- ☐ Pull your hair
- ☐ Scratch yourself
- ☐ Prevent wounds healing
- ☐ Ingest toxic substances
- ☐ Break bones
- ☐ Other

If other please specify ____________________________________

9. How old were you when you first started self-harming?

____

10.	Can you describe what happened the first time you self-harmed?
11.	What factors do you think contributed to you starting to self-harm?

Section C – Your use of websites

The third part of this questionnaire will ask you questions about your use of self-harm forums/websites.

12. When did you first look at deliberate self-harm websites?

- ☐ Before you started self-harming
- ☐ Within 1 month of starting self-harming
- ☐ Within 2 months of starting self-harming
- ☐ Between 2 months and 1 year of starting self-harming
- ☐ After self-harming for longer than one year
- ☐ I have never self-harmed

13.	Why did you first explore deliberate self-harm forums/websites?

14.	What were you looking for from forums/websites when you first started exploring these sites?

15.	Please list the sites you use for forum participation or information.<br>(Forum participation means posting or reading posts in chat rooms or on message boards)

16.	Why do you use these sites over others?

17. How often in an average week would you spending looking at or using forums/websites?

☐ Daily (for longer than 4 hours per day)
☐ Daily (but less than 4 hours per day)
☐ 2-6 times per week
☐ Once a week
☐ Less than once a week but more than once a month
☐ Once a month
☐ Less than once a month

18. What do you do when you use these websites? Tick all that apply:
☐ Search for information

☐View threads in the forum
☐ Contribute to threads in the forum
☐Start threads in the forum
☐Chat
☐Send private messages
☐Play games
☐ Other – please explain

____

19.	Can you explain in your own words why you use these forums/websites?

<u>If you said earlier that you do NOT currently self-harm, please click here to finish the questionnaire and submit your responses [</u>[Directed to end of survey]

<u>If you said earlier that you DO currently self-harm, please click here to continue. There are 3 further questions for you to answer</u> [Directed to Section D]

Section D – How websites influence your behaviour

The fourth part of this questionnaire is to find out if using these forums/websites have affected your behaviour and in what ways.

20.	How has your self-harm behaviour changed since you started using these forums/websites?

21.	Has participating in or viewing these sites affected you in any other way?

Section E – Your beliefs regarding the role of such websites

The final part of this questionnaire is to find out what you think about self-harm forums/websites and how you would explain the sites to other people.

22.	How would you describe the website you most commonly use/explain what the website you most commonly use is for to someone who does not self-harm?

You have now reached the end of the questionnaire.
Thank you very much for taking the time the answer the questions, your responses are much appreciated.
Support can be found at http://www.mind.org.uk/help/diagnoses_and_conditions/self-harm
