# Supplementary material for: Exploring the Use and Effects of Deliberate Self-Harm Websites: An Internet-Based Study
Source: J Med Internet Res. 2013 Dec 20;15(12):e285. doi: 10.2196/jmir.2802 (PMC3875893; doi:10.2196/jmir.2802)
Supplement: Supplementary file 2 [file jmir_v15i12e285_app2.pdf]

Appendix 1 – Websites that hosted the link to the questionnaire

- http://www.lifesigns.org.uk
- http://www.selfharm.co.uk
- http://www.mind.org.uk/help/diagnoses_and_conditions/self-harm
- http://health.groups.yahoo.com/group/secretcutters
- http://www.sirius-project.org
- http://self-injury.net
- http://buslist.org/injury.html
- http://www.thesite.org/healthandwellbeing/mentalhealth/selfharm
- http://www.crazyboards.org/forums/
- http://www.teenhelp.org
- http://ehealthforum.com/health/health_forums
- http://www.patient.co.uk
- http://www.oxfordmhf.org.uk
- http://www.prettythin.com
- http://www.pandys.org
- http://www.tumblr.com
- http://www.psychforums.com
- http://www.mentalhealthforum.net
- http://www.thestudentroom.co.uk
- http://www.recoveryourlife.com
