# Supplementary material for: Exploring the Use and Effects of Deliberate Self-Harm Websites: An Internet-Based Study
Source: J Med Internet Res. 2013 Dec 20;15(12):e285. doi: 10.2196/jmir.2802 (PMC3875893; doi:10.2196/jmir.2802)
Supplement: Supplementary file 3 [file jmir_v15i12e285_app3.pdf]

Appendix 3 – Analysis maps

For the analysis maps shown below, the topic addressed is represented in grey boxes, the themes in black boxes and the coding categories in white boxes.

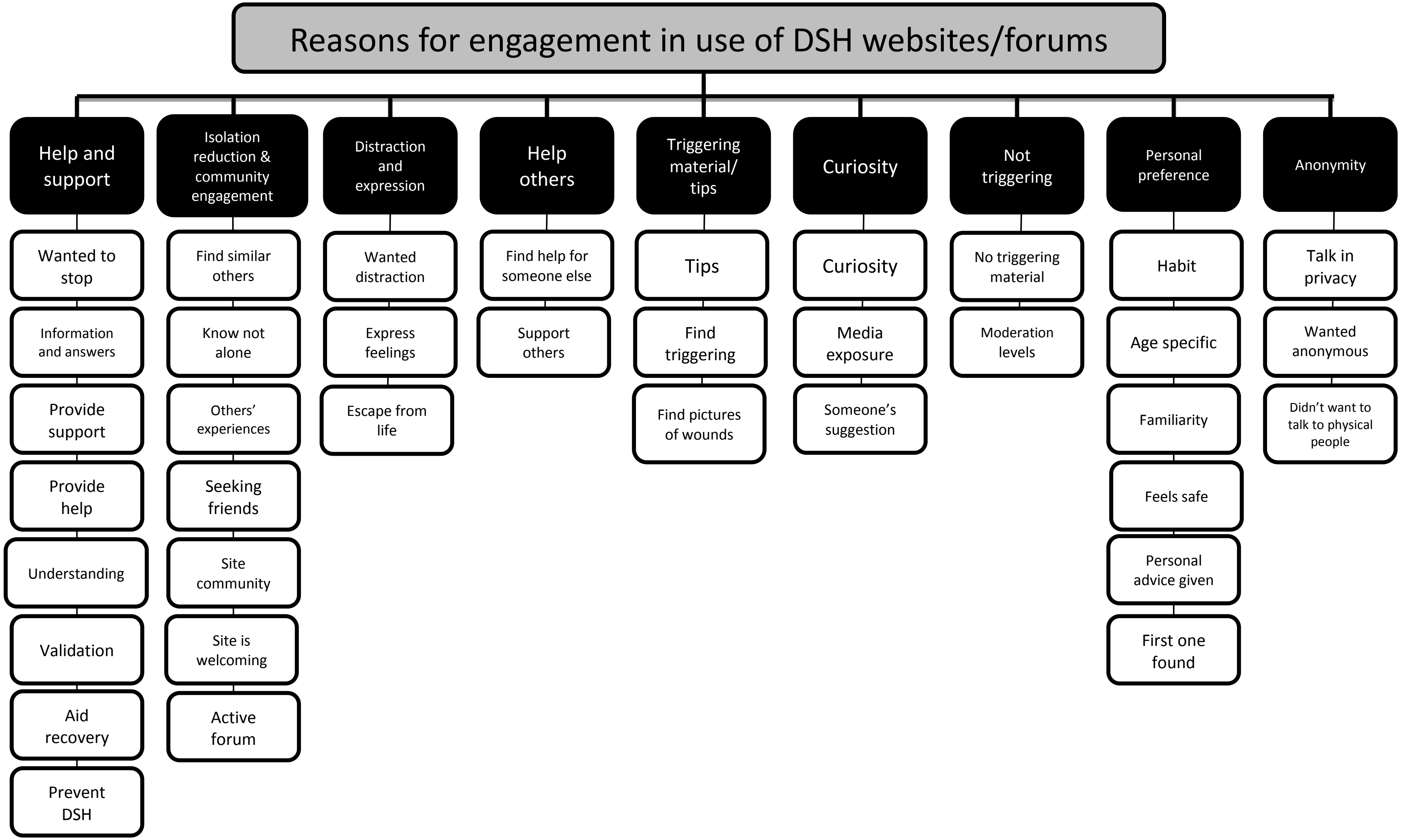

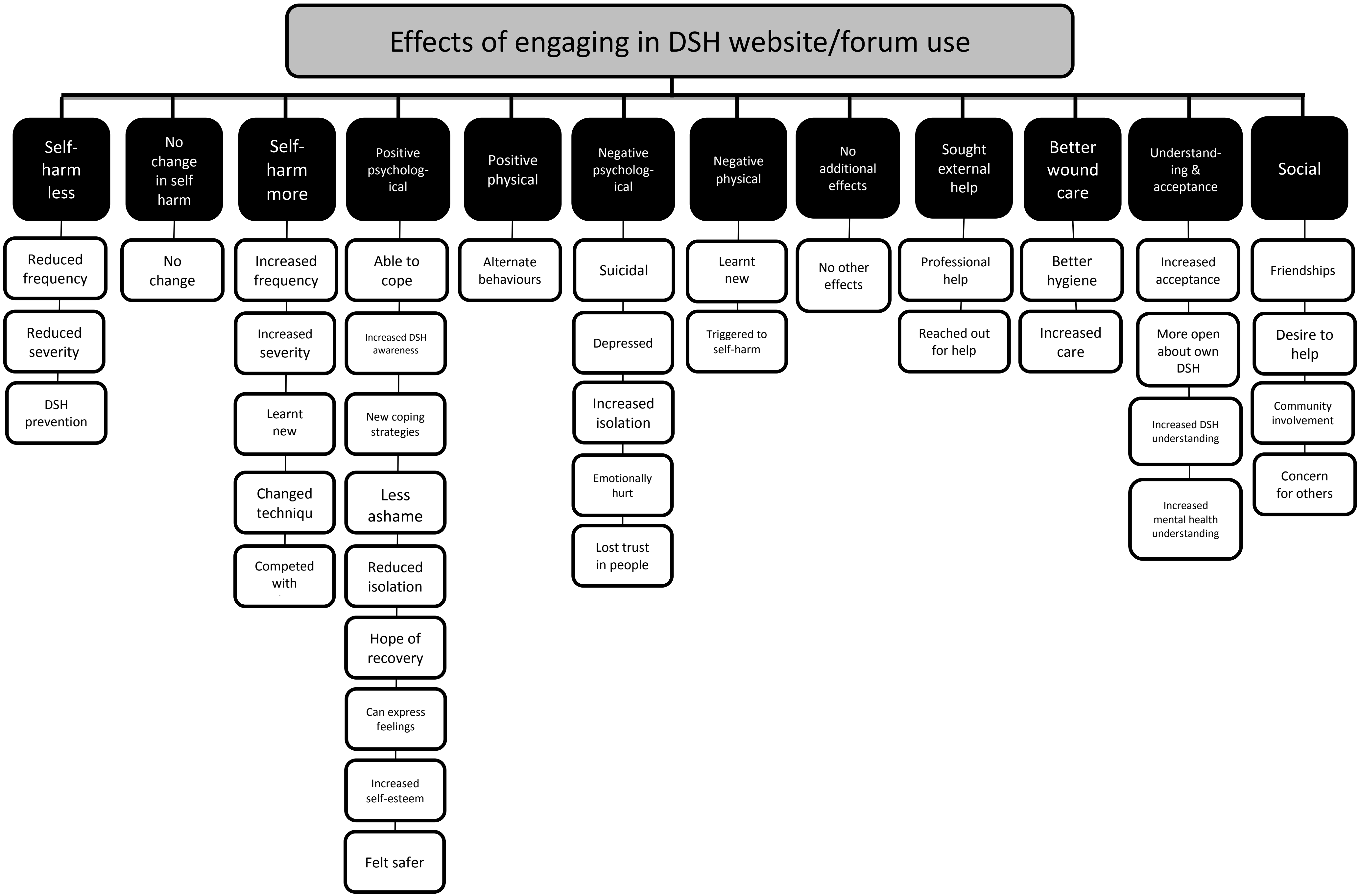
Effects of engaging in DSH website/forum use
Self-harm less
Reduced frequency
Reduced severity
DSH prevention
No change in self harm
No change
Self-harm more
Increased frequency
Increased severity
Learnt new
Changed techniqu
Competed with
Positive psycholog-ical
Able to cope
Increased DSH awareness
New coping strategies
Less ashame
Reduced isolation
Hope of recovery
Can express feelings
Increased self-esteem
Felt safer
Positive physical
Alternate behaviours
Negative psycholog-ical
Suicidal
Depressed
Increased isolation
Emotionally hurt
Lost trust in people
Negative physical
Learnt new
Triggered to self-harm
No additional effects
No other effects
Sought external help
Professional help
Reached out for help
Better wound care
Better hygiene
Increased care
Understand-ing & acceptance
Increased acceptance
More open about own DSH
Increased DSH understanding
Increased mental health understanding
Social
Friendships
Desire to help
Community involvement
Concern for others

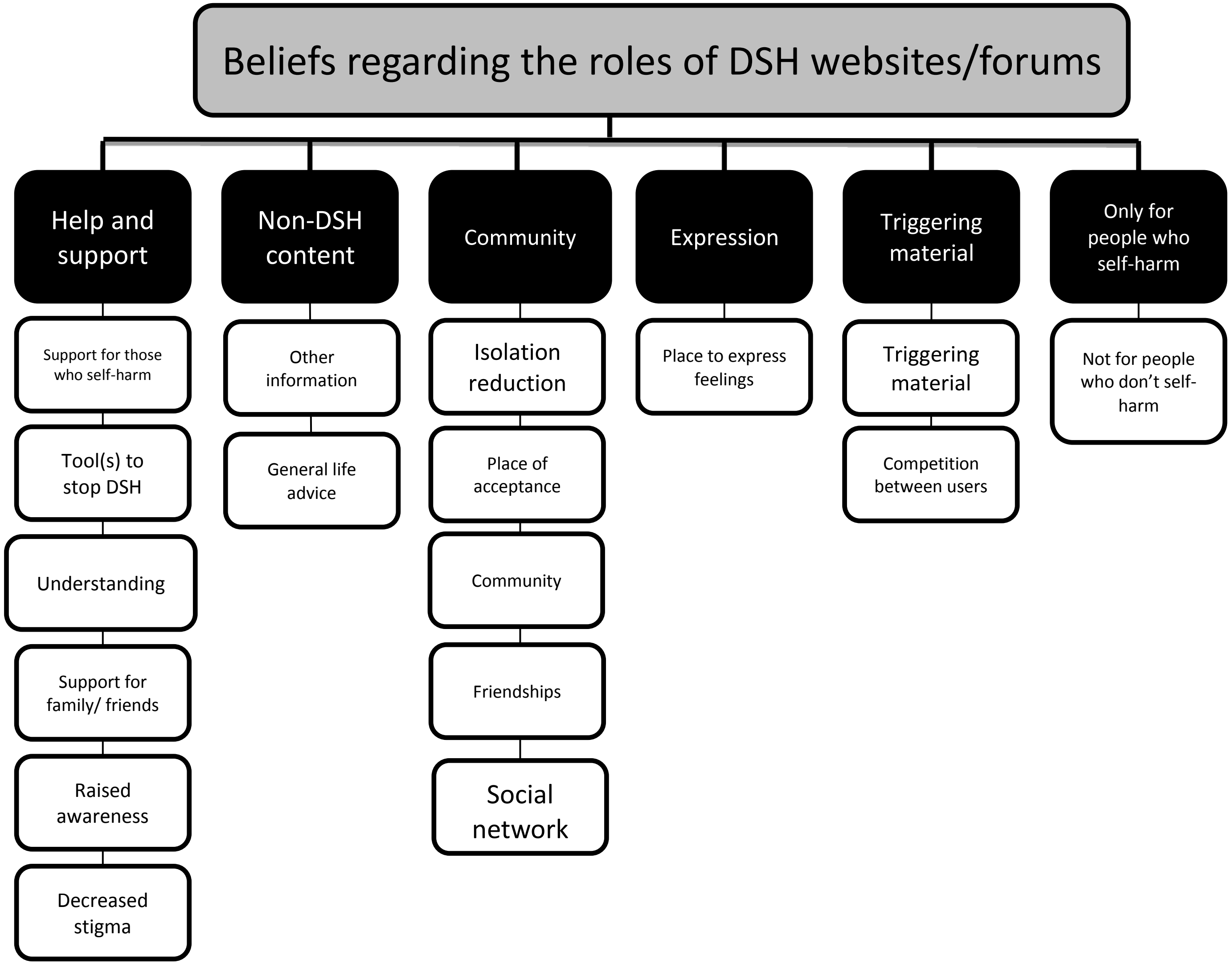

Beliefs regarding the roles of DSH websites/forums
Help and support
Support for those who self-harm
Tool(s) to stop DSH
Understanding
Support for family/ friends
Raised awareness
Decreased stigma
Non-DSH content
Other information
General life advice
Community
Isolation reduction
Place of acceptance
Community
Friendships
Social network
Expression
Place to express feelings
Triggering material
Triggering material
Competition between users
Only for people who self-harm
Not for people who don't self-harm
